# Modulation of hepatitis B virus infection by epidermal growth factor secreted from liver sinusoidal endothelial cells

**DOI:** 10.1038/s41598-020-71453-5

**Published:** 2020-09-01

**Authors:** Shin-Wei Chen, Misao Himeno, Yuta Koui, Masaya Sugiyama, Hironori Nishitsuji, Masashi Mizokami, Kunitada Shimotohno, Atsushi Miyajima, Taketomo Kido

**Affiliations:** 1 Institute for Quantitative Biosciences, The University of Tokyo, 1-1-1 Yayoi, Bunkyo-ku, Tokyo, 113-0032 Japan; 2 Genome Medical Science Project, National Center for Global Health and Medicine, 1-7-1 Kohnodai, Ichikawa, 272-8516 Japan

**Keywords:** Induced pluripotent stem cells, Hepatocytes, Pluripotent stem cells

## Abstract

Hepatocytes derived from human iPSCs are useful to study hepatitis B virus (HBV) infection, however infection efficiency is rather poor. In order to improve the efficiency of HBV infection to iPSC-derived hepatocytes, we set a co-culture of hepatocytes with liver non-parenchymal cells and found that liver sinusoidal endothelial cells (LSECs) enhanced HBV infection by secreting epidermal growth factor (EGF). While EGF receptor (EGFR) is known as a co-receptor for HBV, we found that EGF enhanced HBV infection at a low dose of EGF, whereas EGF at a high dose suppressed HBV infection. EGFR is internalized by clathrin-mediated endocytosis (CME) and clathrin-independent endocytosis (CIE) pathways depending on the dose of EGF. At a high dose of EGF, the endocytosed EGFR via CIE is degraded in the lysosome. This study is the first to provide evidence that HBV is endocytosed via CME and CIE pathways at a low and high dose of EGF, respectively. In conclusion, we developed an in vitro system of HBV infection using iPSC-derived liver cells, and show that EGF secreted from LSECs modulates HBV infection in a dose dependent manner.

## Introduction

Hepatitis B virus (HBV) infection is a significant global public health issue. More than 400 million people in the world are infected with HBV, leading to 1 million annual deaths due to hepatocellular carcinoma, HCC. HBV is a partially double-stranded DNA virus and a member of the *Hepadnaviridae* family of viruses. HBV enters hepatocytes via sodium-taurocholate cotransporting polypeptide (NTCP)^1^ and Epidermal growth factor receptor (EGFR) is known to be involved in NTCP-mediated HBV entry^2^. After entry, HBV establishes a nuclear pool of episomal covalently closed circular DNA (cccDNA), which is then copied into RNA. The viral RNA is transported to the cytoplasm where it is reverse transcribed into DNA and packed in a virus particle^3–6^. This complicated life cycle is a major problem to develop effective antivirus therapeutics. Development of antiviral therapeutics requires an in vitro infection system that faithfully recapitulates the complicated viral life cycle. Primary human hepatocytes have been used for studies on HBV infection, however, these cells are phenotypically unstable and the availability is limited.

Because there are many genotypes of HBV and genetic backgrounds of individuals affect infection of each HBV genotypes, it would be beneficial to generate hepatocytes from human induced pluripotent stem cells (hiPSCs) with different genetic backgrounds^7^. However, hiPSC-derived hepatocytes are immature and exhibit limited capacity for the infection and replication of HBV. We have previously reported that hiPSC-derived non-parenchymal cells (NPCs), liver sinusoidal endothelial cells (LSECs) and hepatic stellate cells (HSCs), promoted maturation of hiPSC-derived hepatocytes^8,9^. In the present study, we developed a trans-well co-culture system of hiPSC-derived liver cells that can be infected with HBV efficiently. We found that LSECs secreted EGF and that EGF modulated HBV infection dose dependently via EGFR-mediated endocytosis pathways.

## Results

### Development a co-culture system of iPSC-derived hepatocytes with NPCs

**Figure 1:**
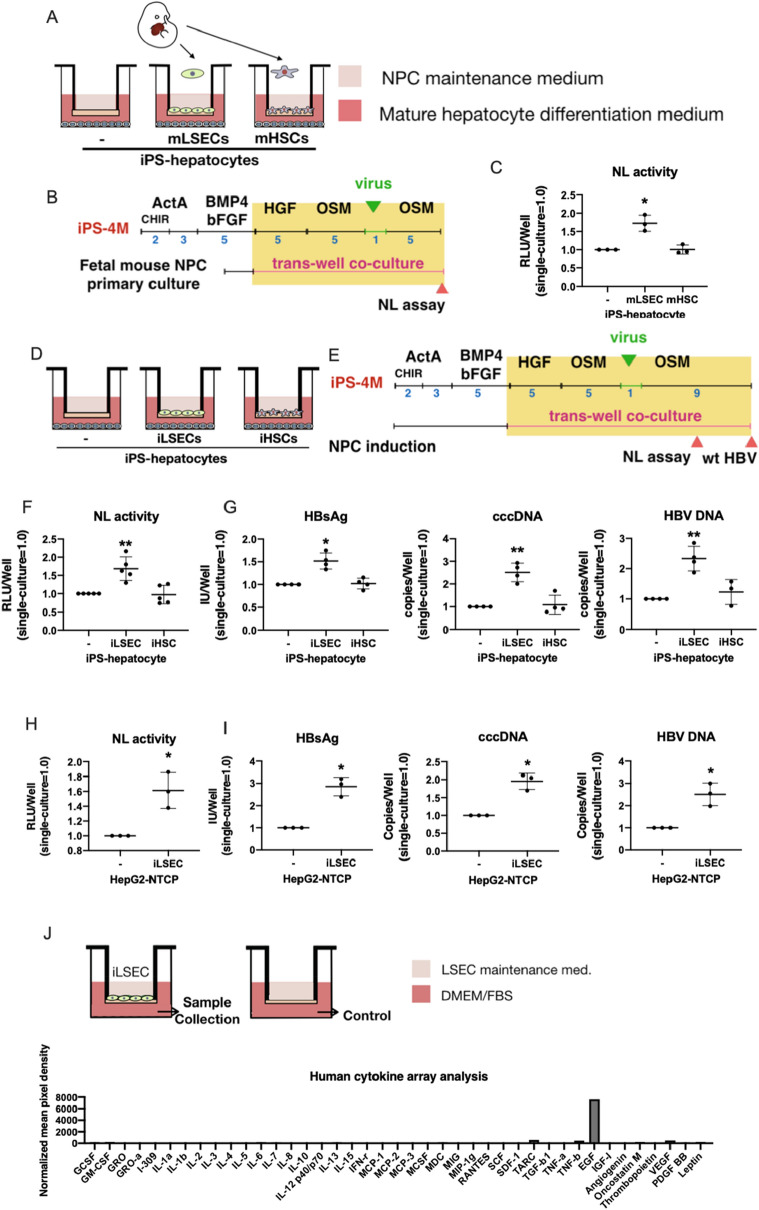
Enhancement of HBV infection to iPSC-derived hepatocytes and HepG2-NTCP cells by fetal mouse LSECs and iPSC-derived LSECs. (**A**) Trans-well co-culture system of iPSC-derived hepatocytes with fetal mouse NPCs. (**B**) Schematic representation of the HBV/NL infection assay in iPSC-derived hepatocytes co-cultured with fetal mouse NPCs. (**C**) Relative HBV/NL activities in iPSC-derived hepatocytes (−), iPSC-derived hepatocytes co-cultured with fetal mouse LSECs (LSEC), and fetal mouse HSCs (HSC). The results are shown as the mean ± SEM of 3 independent experiments. The significant difference was determined in each group compared with the control group. *p < 0.05. (**D**) Trans-well co-culture system of iPSC-derived hepatocytes with iPSC-derived NPCs. (**E**) Schematic representation of the HBV/NL and wild type HBV infection assay in iPSC-derived hepatocytes co-cultured with iPSC-derived NPCs. (**F**) Relative HBV/NL activities in iPSC-derived hepatocytes (–), iPSC-derived hepatocytes co-cultured with iPSC-derived LSECs (iLSEC), and iPSC-derived HSCs (iHSC). The results are shown as the mean ± SEM of 5 independent experiments. The significant difference was determined in each group compared with the control group. **p < 0.01. (**G**) Levels of HBsAg, cccDNA and HBV DNA in iPSC-derived hepatocytes (−), iPSC-derived hepatocytes co-cultured with iPSC-derived LSECs (iLSEC), and iPSC-derived HSCs (iHSC). The results are shown as the mean ± SEM of 4 independent experiments. The significant difference was determined in each group compared with the control group. *p < 0.05, **p < 0.01. (**H**) Relative HBV-NL activities in HepG2-NTCP cells (–) and HepG2-NTCP cells co-cultured with iPSC-derived LSECs (iLSEC). The result is shown as the mean ± SEM of 3 independent experiments. *p < 0.05. (**I**) Levels of HBsAg, cccDNA and HBV DNA in HepG2-NTCP cells (−) and HepG2-NTCP cells co-cultured with iPSC-derived LSECs (iLSEC). The results are shown as the mean ± SEM of 3 independent experiments. *p < 0.05. (**J**) Detection of EGF in the supernatant of iPSC-derived LSECs by human cytokine array analysis. Trans-well inserts without iPSC-derived LSECs was used as the control. Normalized mean pixel density = mean pixel density on sample array × positive control on sample array/positive control on control array–mean pixel density on control array.

Hepatocytes derived from iPSCs are immature and we previously showed that NPCs promote maturation of hepatocytes in vitro^9^. We therefore tested if HBV infection is enhanced by NPCs. First, we isolated and cultured LSECs and HSCs from E14.5 mouse fetal livers as Stab2^+^ and Ngfr^+^ cells, respectively, which exhibited distinctive phenotypes (Supplementary Fig. 1A–E). They were co-cultured with hiPSC-derived hepatocytes by using a Transwell cell culture insert (Fig. 1A). HBV infection was evaluated by a recombinant hepatitis B virus (HBV) expressing NanoLuc (NL) (HBV/NL)^10^ (Fig. 1B). Interestingly, LSECs significantly enhanced NL activity after 5 days of virus infection, whereas HSCs did not enhance it (Fig. 1C). The results indicated that the co-culture system of iPSC-derived hepatocytes with NPCs would be useful for the establishment of HBV infection model and the studies on the mechanisms of the infection and replication of HBV.

As we have already established culture systems to generate LSECs and HSCs from hiPSCs and shown that they promote hepatic maturation of hiPSC-derived hepatocytes in vitro^9^, we applied those hiPSC-derived NPCs to HBV infection (Fig. 1D). After induction of hepatic endodermal cells from iPSCs, they were co-cultured with hiPSC-derived LSECs or HSCs in trans-well cell culture inserts and infected with HBV/NL virus or wild-type HBV (Fig. 1E). Similar to fetal mouse LSECs, hiPSC-derived LSECs also significantly enhanced HBV infection in the trans-well co-culture system as shown by NL activity, HBsAg, cccDNA, and HBV DNA (Fig. 1F,G). Thus, we established a HBV infection model by using iPSC-derived liver cells. These data demonstrated that the co-culture system of hiPSC-derived liver cells would be useful for studies on relationships between genotypes of HBV and genetic backgrounds of individuals.

### Mechanism of the enhancement of HBV infection by LSECs

To reveal how hiPSC-derived LSECs enhance the HBV infection and replication, we utilized the HepG2-NTCP hepatoma cell line overexpressing NTCP, which provides a simplified system to evaluate HBV infection^10^ (Supplementary Fig. 2A,B). In the trans-well co-culture system, hiPS-derived LSECs significantly enhanced NL activity, HBsAg, cccDNA, and HBV DNA (Fig. 1H,I), showing that the simplified culture system can be used to study HBV infection and replication. Because the trans-well co-culture system avoids a direct cell–cell contact, the positive effect of LSECs on HBV infection should be mediated by a soluble factor. We therefore performed cytokine array analysis of LSEC culture medium to find a factor responsible for enhancing HBV infection. Among 42 cytokines we analyzed, EGF was found to be most abundant in the supernatant of iPSC-derived LSECs (Fig. 1J). In contrast, no detectable EGF was found in the supernatant from iPS-derived HSCs, indicating that EGF from LSECs regulated the HBV infection in the co-culture model.

### Modulation of HBV infection by EGF

**Figure 2:**
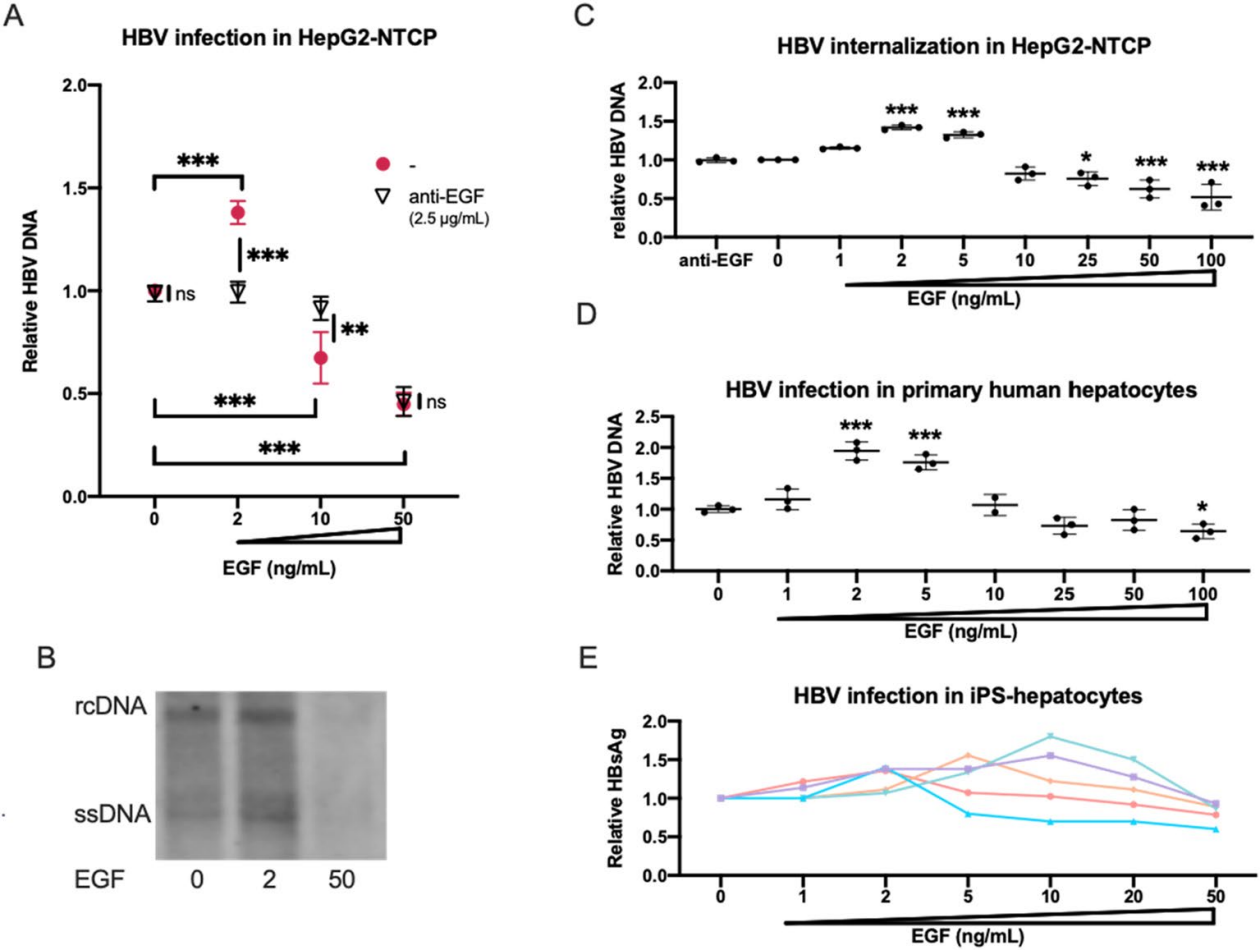
Modulation of HBV infection by EGF. (**A**) Relative HBV DNA level in HepG2-NTCP cells at 2 ng/ml, 10 ng/ml and 50 ng/ml of EGF with/without 2.5 µg/ml anti-EGF antibody. The result is shown as the mean ± SEM of 3 independent experiments. **p < 0.01, ***p < 0.001. (**B**) Southern blot analysis of HBV DNA fractions obtained from HepG2-NTCP cells infected with HBV at post-infection day 7. (**C**) Relative HBV DNA level in HepG2-NTCP cells at 0 ng/ml, 1 ng/ml, 2 ng/ml, 5 ng/ml, 10 ng/ml, 25 ng/ml, 50 ng/ml and 100 ng/ml of EGF in HBV internalization assay. The result is shown as the mean ± SEM of 3 independent experiments. The result is shown as the mean ± SEM of 3 independent experiments. The significant difference was determined in each group compared with the control group. *p < 0.05, ***p < 0.001. (**D**) Relative HBV DNA level in primary human hepatocytes at 0 ng/ml, 1 ng/ml, 2 ng/ml, 5 ng/ml, 10 ng/ml, 25 ng/ml, 50 ng/ml and 100 ng/ml of EGF. The result is shown as the mean ± SEM of 3 samples in one experiment. The significant difference was determined in each group compared with the control group. *p < 0.05, ***p < 0.001. (**E**) Relative HBsAg level in iPS-hepatocytes at 0 ng/ml, 1 ng/ml, 2 ng/mL, 5 ng/ml, 10 ng/ml, 20 ng/ml, and 50 ng/ml of EGF in HBV infection assay. The result is shown as 5 independent experiments.

It was recently reported that the EGFR activation by EGF plays a positive role for NTCP-mediated HBV internalization^2^. To confirm the enhancement of HBV infection by EGF, we performed HBV infection assay using HepG2-NTCP at 2 ng/ml, 10 ng/ml and 50 ng/ml of EGF. HBV infection was enhanced by EGF at 2 ng/ml. Unexpectedly, however, HBV infection was down-regulated at a higher concentration of EGF (Fig. 2A,B, Supplementary Fig. 3). They were neutralized by the treatment of neutralizing anti-EGF antibody (Fig. 2A). We also found that HBV infection was down-regulated in HepG2-NTCP when co-cultured with a high density of hiPSC-derived LSECs (data not shown), suggesting that EGF at a high dose suppressed HBV infection. This EGF-dependent modulation of HBV internalization occurred before 24 h of post-infection (Fig. 2C). These results were confirmed by using primary human hepatocytes and iPSC-derived hepatocytes. In primary human hepatocytes, 2 ng/ml of EGF enhanced HBV infection in the FBS-free condition and the infection was gradually reduced over 5 ng/ml of EGF (Fig. 2D). As this EGF dose response curve is related with cell density (data not shown), and it is difficult to be controlled in iPSC-derived hepatocytes, it showed various patterns to EGF; however, dose-dependent regulation was reproducibly observed (Fig. 2E). These results suggested that modulation of the HBV infection by EGF was dose-dependent.

### Activated EGFR enhance HBV attachment

**Figure 3:**
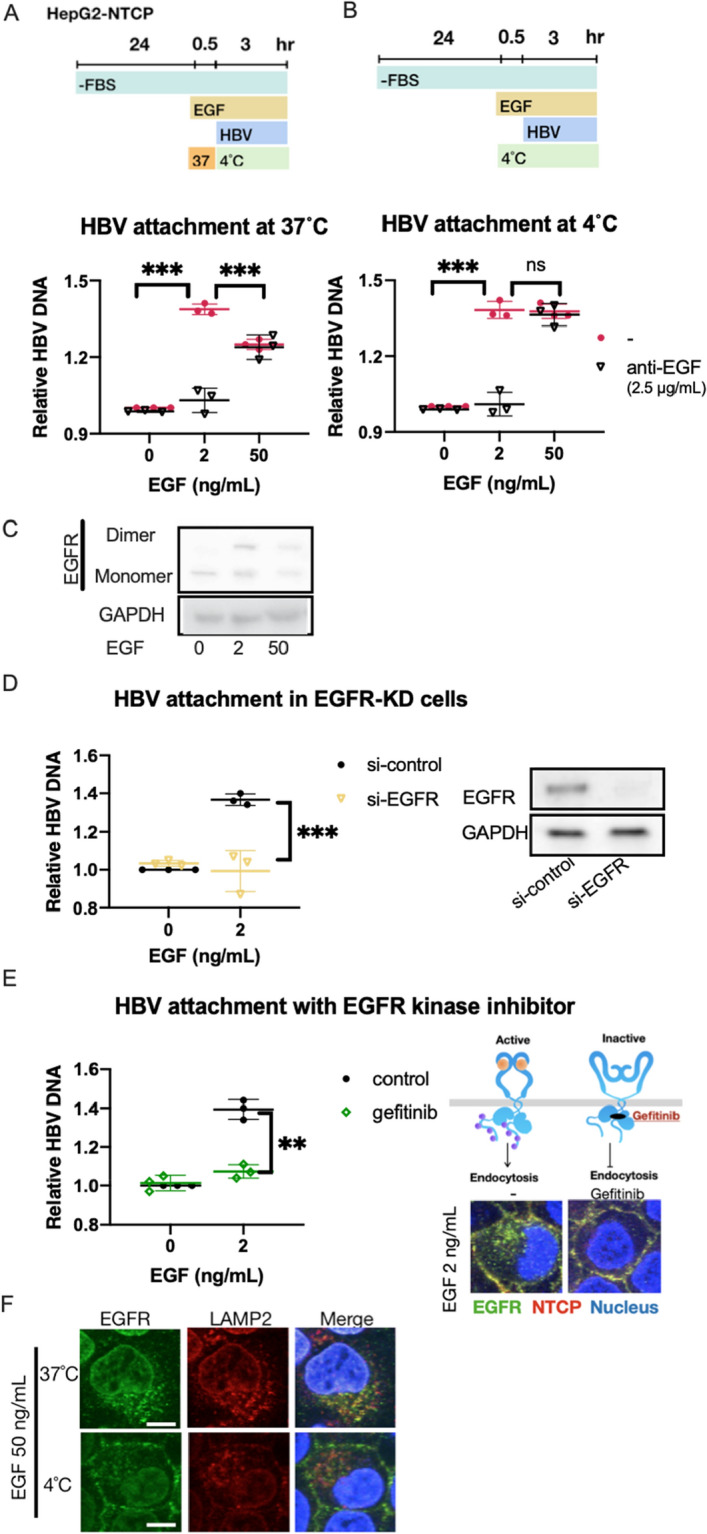
Attachment of HBV by stimulation of EGF. (**A**,**B**) Relative HBV attachment in HepG2-NTCP cells treated with 2 ng/ml and 50 ng/ml of EGF at 37 °C (**A**) and 4 °C (**B**). The results are shown as the mean ± SEM of 3 independent experiments. ***p < 0.001. N.S., not significant. (**C**) Western blot analysis of cell surface protein treated with cross-linker BS^3^ at 0 ng/ml, 2 ng/ml and 50 ng/ml. The same amount of cell lysates was loaded in each lane, treated with anti-EGFR antibody and anti-GAPDH antibody, respectively. EGFR monomer: 175 kDa, EGFR dimer: 350 kDa. (**D**) Relative HBV attachment in EGFR knockdown HepG2-NTCP cells pretreated with 2 ng/ml of EGF. The result is shown as the mean ± SEM of 3 independent experiments. ***p < 0.001. The same amount of cell lysates was loaded in each lane, treated with anti-EGFR antibody and anti-GAPDH antibody, respectively. (**E**) Relative HBV attachment in HepG2-NTCP cells treated with or without 10 µM gefitinib and 2 ng/ml EGF at 4 °C. The results are shown as the mean ± SEM of 3 independent experiments. **p < 0.01. ***p < 0.001. Immunofluorescence staining for EGFR (green) and NTCP (Red) in Hepg2-NTCP cells treated with/without gefitinib at 2 ng/ml of EGF at 37 °C and 4 °C. (**F**) Immunofluorescence staining for EGFR (green) and LAMP2 (Red) in Hepg2-NTCP cells at 50 ng/ml of EGF at 37 °C and 4 °C. Scale bar, 10 µm.

To reveal whether EGF affected HBV infection at the cell surface or intracellular pathways, we examined the attachment of HBV to HepG2-NTCP cells. When the cells were pretreated with EGF at 37 °C for 30 min, EGF at 2 ng/ml enhanced the binding of HBV to the cells, which can be totally inhibited by neutralizing anti-EGF antibody (2.5 µg/ml). Interestingly, the enhancement by 50 ng/ml EGF was weaker than that by 2 ng/ml (Fig. 3A). By contrast, EGF enhanced the HBV binding equally well when pretreated at 4 °C to prevent EGFR internalization (Fig. 3B). From the western blot analysis using cross-linker BS^3^, the cell surface EGFR existed as a monomeric form without EGF stimulation, and both monomer and dimer forms were found in the presence of EGF; at a high dose of EGF, less EGFR was detected compared to a low dose of EGF (Fig. 3C, Supplementary Fig. 3). Knockdown of EGFR expression cancelled the enhanced HBV attachment by EGF (Fig. 3D, Supplementary Fig. 3), suggesting that EGF stimulation induced the HBV attachment. To confirm this possibility, we used an EGFR kinase inhibitor, gefitinib^11,12^, and found that gefitinib also reversed the enhanced HBV attachment by EGF (Fig. 3E), indicating that activated EGFR enhanced HBV attachment. To reveal the mechanisms of the differential response to 2 and 50 ng/ml of EGF at 37 °C, we performed immunocytochemical analysis and found that few EGFR was colocalized with a lysosome maker, LAMP2, whereas EGFR was mostly expressed on the cell membrane at 4 °C compared with 37 °C (Fig. 3F). As the receptor internalization was suppressed at 4 °C, the results suggested that the effect of EGF on HBV binding to the cell surface was independent from EGF doses, and that the dose-dependent effect of EGF on HBV infection was caused by an event post binding to cells.

### Endocytosis of EGFR and HBV

**Figure 4:**
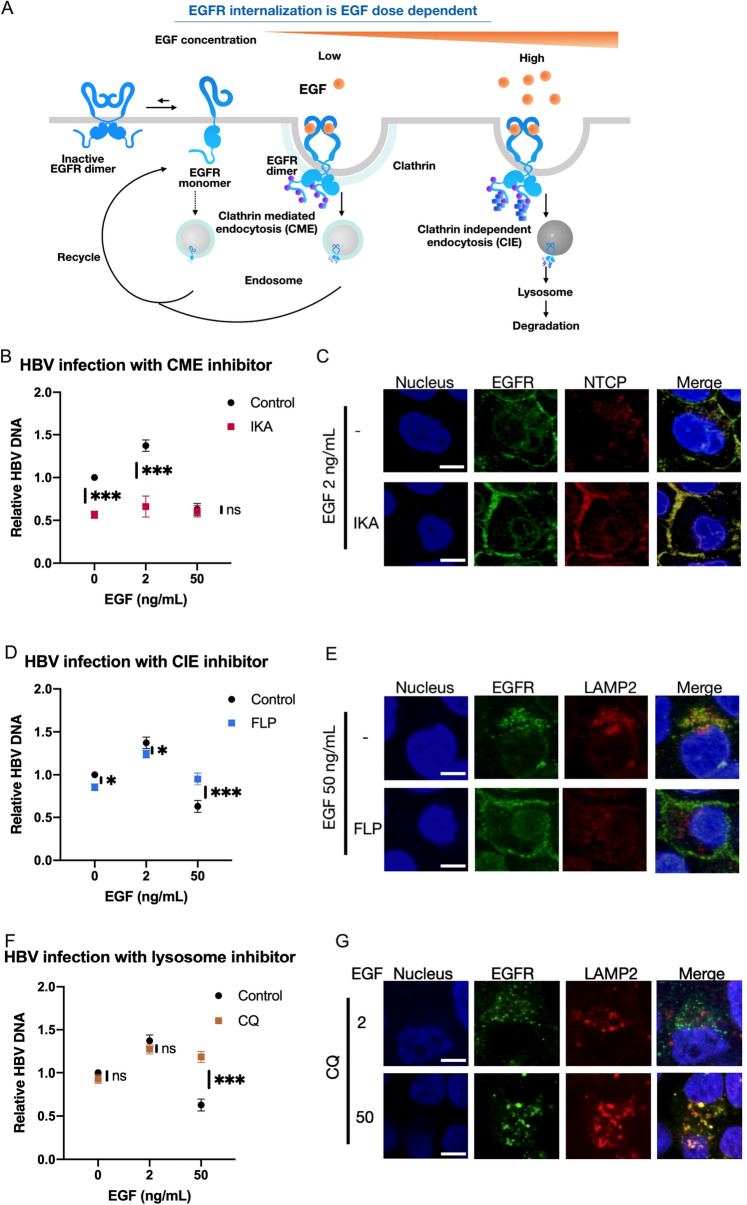
HBV is internalized via the EGFR endocytosis pathways. (**A**) The dose-dependent EGFR endocytosis pathway modulated by EGF. The illustration of EGFR monomer and dimer were referred to previous report^23^. (**B**,**D**,**F**) Relative HBV infection levels in HepG2-NTCP cells (−), HepG2-NTCP cells treated with ikarugamycin (IKA) (**B**), filipin (FLP) (**D**) and chloroquine (CQ) (**F**). The results are shown as the mean ± SEM of 3 independent experiments. *p < 0.05. **p < 0.01. ***p < 0.001. N.S., not significant. (**C**) Immunofluorescence staining for EGFR (green) and NTCP (Red) in HepG2-NTCP cells at 2 ng/ml of EGF treated with or without 2 µM IKA. Scale bar, 10 µm. (**E**) Immunofluorescence staining for EGFR (green) and LAMP2 (Red) in HepG2-NTCP cells at 50 ng/ml of EGF treated with or without 1 µM FLP. Scale bar, 10 µm. (**F**) Immunofluorescence staining for EGFR (green) and LAMP2 (Red) in HepG2-NTCP cells at 2 ng/ml and 50 ng/mL of EGF treated with 25 µM CQ. Scale bar, 10 µm.

Previous studies showed that the internalization of EGFR was EGF dose-dependent via different endocytosis pathways^13,14^. At a low dose of EGF stimulation, activated EGFR is internalized via clathrin-mediated endocytosis (CME) and then recycled back to the cell surface. On the other hand, the activated EGFR by a high dose of EGF is internalized through clathrin-independent endocytosis (CIE), which is initiated by a higher level of phosphorylation at the C-terminal of EGFR. Ubiquitin is added to the highly phosphorylated EGFR, which is transported to the late endosome and subsequently to the lysosome to be degraded (Fig. 4A). Therefore, we hypothesized that HBV was internalized with EGFR via the CIE pathway at the high dose of EGF and degraded. To test this possibility, we evaluated the effect of specific inhibitors to block each pathway, CME and CIE. Ikarugamycin (IKA)^15^, a CME inhibitor, blocked HBV infection at 0 ng/ml and 2 ng/ml of EGF, whereas it had no effect on HBV infection at 50 ng/ml of EGF (Fig. 4B). Immunocytochemistry showed IKA blocked the internalization of both EGFR and NTCP (Fig. 4C). On the other hand, the suppression of HBV infection by the high dose of EGF was reversed by the treatment with filipin (FLP), a CIE inhibitor^14^ (Fig. 4D). Immunocytochemical analysis showed the colocalization of EGFR and LAMP2, a lysosome marker, by stimulation with EGF at a high dose. And the colocalization was inhibited by FLP (Fig. 4E), suggesting that HBV was degraded via the CIE pathway. We investigated this possibility by inhibiting lysosomal functions. As expected, HBV infection was clearly increased in the presence of chloroquine (CQ), a lysosomal inhibitor, at 50 ng/ml of EGF^16,17^ (Fig. 4F). The colocalization of EGFR and LAMP2 was only observed at a high dose but not low dose (Fig. 4G). Taken together, these results strongly suggested that HBV is internalized by the pathways shared with EGFR endocytosis, and that HBV is degraded in the lysosomes at a high dose of EGF.

## Discussion

Primary cultures of human hepatocytes have been used for HBV infection assays in vitro. However, the supply of fresh human hepatocytes is limited. Hepatocytes derived from iPSCs will be a stable source for HBV infection system, which will also provide a tool to study the genetic relationship between HBV genotype and susceptive individuals. However, the hepatocytes derived from iPSCs are immature and HBV infection efficiency is limited. During liver development, immature hepatocytes proliferate and differentiate into mature hepatocytes through interactions with hepatic NPCs such as LSECs and HSCs. NPCs secrete factors such as HGF, EGF, BMP and FGF that play a central role in liver development. Accordingly, we established a trans-well co-culture system of iPS-derived hepatocytes with NPCs to improve the hepatic maturation by a paracrine mechanism. Previously, we found that the HSCs support maturation of iPSC-derived hepatocytes. Surprisingly, we found in this study that LSECs (both mouse embryonic LSECs and iPSC-derived LSECs), but not HSCs enhanced the HBV infection, showing that the paracrine regulation for HBV receptor is also an important factor for HBV infection. The co-culture system of iPSC-derived hepatocytes with LSECs will be a useful model to establish the HBV infection model and study the mechanism of HBV infection.

**Figure 5:**
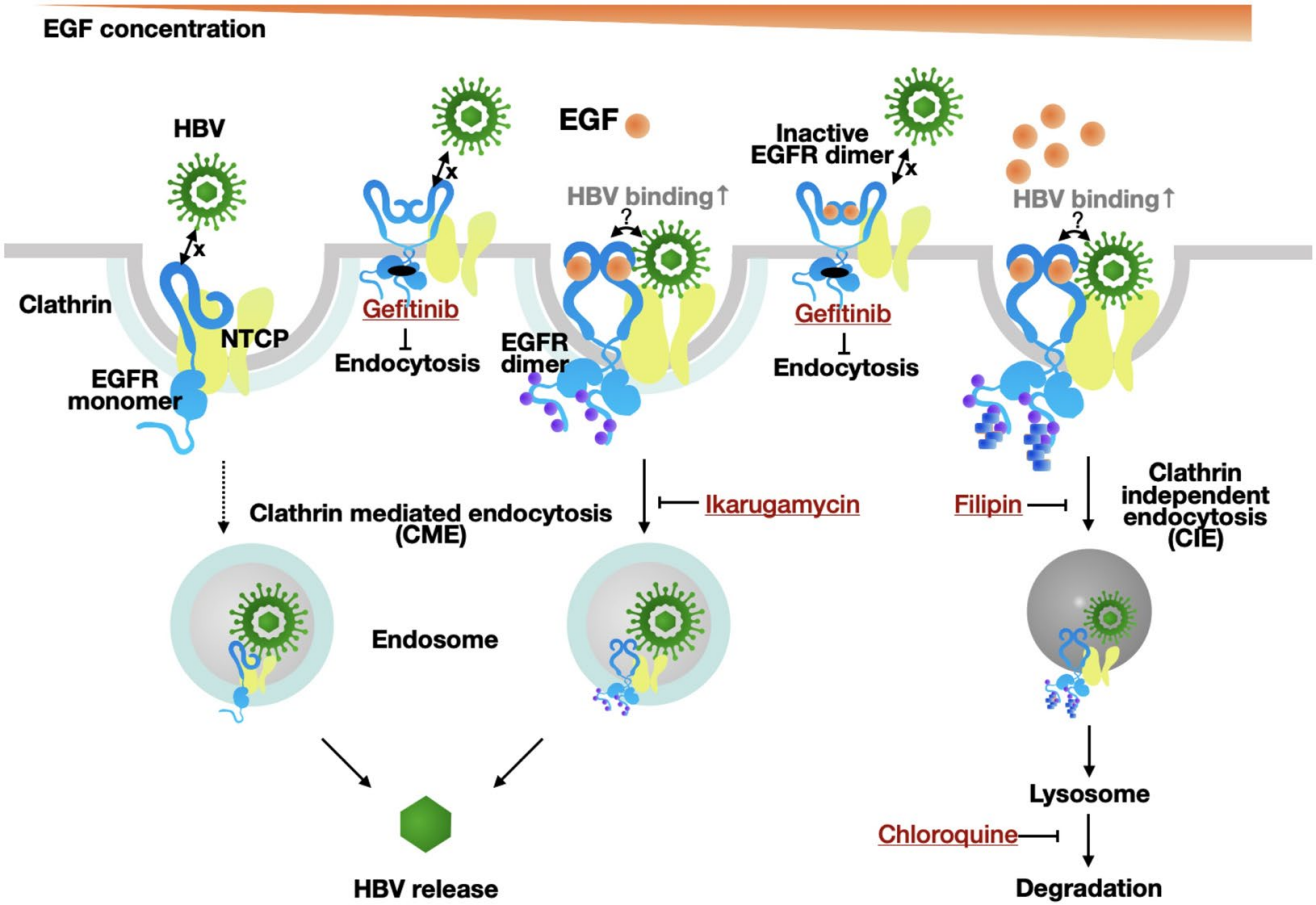
Summary. Modulation of HBV infection by EGF.

Cytokine array analysis revealed that EGF is abundantly secreted from LSECs. While it was recently shown that EGF enhanced HBV infection, an interesting finding in this study is that EGF modulates HBV infection in a dose dependent manner. HBV infection starts by the low affinity interaction with heparin sulfate proteoglycan on the cell surface^18^, followed by high-affinity binding to NTCP. A recent report showed that EGFR is a co-receptor of NTCP and EGF enhances NTCP-mediated HBV infection. Our results also show that EGF enhanced HBV infection. Furthermore, we found that the enhancement was evident at the HBV attachment step, and the enhancing effect of EGF internalization was observed only at a low dose of EGF; EGF at a high dose rather suppressed HBV infection. Activated EGFR is known to be internalized by CME and CIE in a dose dependent manner. At a low dose of EGF, EGFR is internalized via CME to the endosome and recycled back to the cell surface. In contrast, at a high dose, EGFR is highly phosphorylated and ubiquitinated, trafficking to the lysosome to be degraded via the late endosome^14,19^. Using specific inhibitors for CME and CIE, we provide evidence that HBV is endocytosed via CIE by a high dose of EGF (Fig. 5). These results collectively indicate that the EGF concentration is a factor to affect HBV infection to hepatocytes and that it should be considered for optimizing HBV infection in vitro. Moreover, the circulating HBV pass through the fenestrae of LSECs, and enters into a space of dissé, a gap between LSECs and basolateral membrane of hapatocytes^10^ where NTCP is expressed. Our results in vitro that EGF secreted by LSECs regulates the HBV infection suggest a possibility that LSECs also play an important role for HBV infection in vivo.

## Materials and methods

### Cell lines and culture

The hiPSC line TkDN4-M was obtained from the Institute of Medical Science, the University of Tokyo^20^. hiPSCs were maintained in feeder-free culture using Essential 8 Medium or StemFlex medium (Thermo Fisher Sciences). hiPSC-derived LPCs, LSECs, and HSCs were prepared according to previous protocols^7,9^. HepG2-NTCP cells^10^ were cultured in Dulbecco's Modified Eagle Medium (Life Technologies, Carlsbad, CA, USA) supplemented with 10% FBS, MEM non-essential amino acids solution, 100 U/ml penicillin, and 100 µg/ml streptomycin. Primary human hepatocytes (PXB-cells) were purchased from PhoenixBio, maintained in its commercial medium; during HBV infection, FBS and EGF were removed and 4% PEG8000 and 2% DMSO were added to the medium.

### Isolation and culture of fetal mouse liver cells

LSEC and HSC progenitors were isolated from livers of E14.5 fetal mice. The fetal livers (each sample contained 25–35 embryos) were minced and dissociated in Liver Digest Medium (Life Technologies, California, US). Cells were treated with FcR blocking reagent and incubated with FITC-conjugated anti-stab2 antibody and APC-conjugated anti-Ngfr antibody (Miltenyi biotec). Stab2^+^ cells and Ngfr^+^ cells were isolated by a MoFlo XDP cell sorter (Beckman Coulter, Inc, California, US). After sorting, cells were seeded onto a collagen I-coated dish at the density of 40,000 cells/cm^2^ in Endothelial Cell Growth Basal Medium plus (LONZA) supplemented with VEGF (50 ng/ml) and Y27632 (10 μM) under hypoxic condition. Quantitative RT-PCR was performed using SYBR Premix EX TaqII (Takera bio). Primers and antibodies are listed in Supplementary information.

### Endocytosis of EGFR

Cells were seeded at 25,000 cells/cm^2^ and incubated with FBS-starved medium on the next day for 24 h and then stimulated with EGF for 30 min at 37 °C, followed by HBV infection. To inhibit endocytosis, cells were incubated with 2 µM of ikarugamycin (SIGMA) or 1 µg/ml of filipin (SIGMA) for 30 min before EGF (PeproTech) stimulation. For lysosome inhibition, cells were incubated with 25 µM of chloroquine (SIGMA) for 2.5 h before EGF stimulation. To block EGF stimulation, cells were incubated with 10 µM gefitinib (SIGMA) when EGF was added. The negative control for EGF stimulation, EGF neutralizing antibody (Sino biological, 2.5 µg/ml) was used.

### Co-culture system

Hepatocytes derived from iPSCs were in the lower chamber of trans-well. To induce hepatic maturation, cells were incubated in Hepatocyte Basal Medium (LONZA) supplemented with HCM SingleQuots (excluding EGF) and Oncostatin M (20 ng/ml) (PeproTech). NPCs were suspended in Endothelial Cell Growth Basal Medium plus (LONZA) supplemented with VEGF (50 ng/ml) and Y27632 (10 μM) and seeded into the upper chamber of trans-well. Co-culture started at HGF stage. After 10 days of co-culture, cells were infected with HBV for 16 h. HepG2-NTCP cells were suspended in HepG2-NTCP maintenance medium and seeded on a collagen I-coated plate at the density of 25,000 cells/cm^2^. iPSC-derived NPCs were suspended in NPC maintenance medium and seeded into the upper chamber of trans-well. Co-culture started at day 1, and after 6 days of co-culture, cells were infected with HBV for 16 h.

### HBV/NL assay

HBV/NL virus was prepared as previously described^10^. Cells were infected with HBV/NL at 100 GEq/cell in the presence of 4% PEG8000 and 2% DMSO for 16 h. After washing to remove the virus, cells were maintained for 5 days. Activity of NL was then measured using the NL Luciferase Assay Kit (Promega) according to the manufacturers’ protocols.

### HBV infection assay

HBV in the culture supernatant of HepAD38 cells was used as previously described^16^. Medium was harvested and the virus fraction prepared by precipitation with 13% PEG8000 (SIGMA) containing 0.75 M NaCl. Virus was then purified by precipitation at 1,750×*g* for 30 min, suspended in Opti‐MEM (Life Technologies) and stored at − 80 °C until use. Cells were infected with the virus at 10,000 GEq/cell in the presence of 4% PEG8000 and 2% DMSO for 16 h. After washing to remove virus, cells were cultured for 14 days in the medium containing 4% PEG8000 and 2% DMSO. HBsAg was detected by ELISA assay; HBV DNA and cccDNA were detected by qPCR and southern blot^21,22^. Primers and probes are listed in Supplementary information.

### HBV attachment assay

HBV derived from the culture supernatant of HepAD38 cells. Cells were incubated with the virus at 10,000 GEq/cell in the presence of 4% PEG8000 and 2% DMSO at 4 °C for 3 h. After washing out the free virus, HBV DNA was detected by qPCR. Primers are listed in Supplementary information.

### HBV internalization assay

Following HBV attachment assay, the cells were washed to remove free virus and incubated at 37 °C for 24 h. The attached virus was removed by 0.25% trypsin/EDTA and HBV DNA was detected by qPCR.

### Cytokine array analysis

Human Cytokine Antibody Array Membrane (Abcam) was used to detect cytokines according to the manufacturer’s protocol. iPSC-derived LSECs were suspended in NPC maintenance medium and seeded at the density of 40,000 cells/cm^2^ in the upper chamber of trans-well. Control wells contained NPC maintenance medium only. HepG2-NTCP maintenance medium was added to the lower chamber and changed to fresh medium every day. The samples were prepared from the lower chamber at day 5, and the data were analyzed by ImageJ quantitatively. The pictures were taken in ImageQuant LAS 4000 version1.2.

### Cross-linking assay

In cross-linking assay, the cells were incubated with the cross-linker BS^3^ at 4 °C for 30 min, and the cross-linking reaction was terminated using 20 mM of glycine (final concentration) as previously described^11^. Collected and isolated the cell surface lysates using Pierce Cell Surface Protein Isolation Kit (Thermo Fisher Sciences). Samples were then separated on SDS-PAGE, performed western blotting. Antibodies are listed in Supplementary information.

### Knockdown of EGFR

Sequences of siRNAs used for knockdown EGFR are: si-EGFR: 5′- GGAACUGGAUAUUCUGAAA TT-3′ and 5′-GAUCUUUCCUUCUUAAAGA TT-3′.

Cells were transfected with siRNAs at a final concentration of 30 nM using Lipofectamine RNAiMAX Reagent (Life Technologies). The Mission siRNA Universal Negative control were purchased from Sigma-Aldrich (St. Louis, MO, USA). Cells were used for experiments 48 h post-transfection.

### Immunocytochemistry

Cultured cells were fixed in 4% PFA and permeabilized with 0.25% tritonX-100. The cells were blocked with SuperBlock T20 Blocking Buffer (Thermo). Then, they were incubated with primary and secondary antibodies. Antibodies are listed in Supplementary information. The images were taken by confocal microscope and merged by OLYMPUS FV1000 Viewer.

### Statistical analysis

Data are expressed as mean ± SEM and analyzed by Student’s t-test, one-way ANOVA and two-way ANOVA. The statistical significance was determined at P < 0.05.

### Animal experiments and ethics statement

C57BL/6J mice were purchased from CLEA Japan, Inc (Tokyo, Japan). All mouse experiments were approved by the Animal Experiment Ethics Committees at the Institute for Quantitative Biosciences, University of Tokyo (Approval number: 3004, 3105, 0209). Experiments were performed according to the guidelines of the institutional Animal Care and Use Committee of the University of Tokyo.

## Supplementary information

Supplementary Information 1.
