# Supplementary material for: Modulation of hepatitis B virus infection by epidermal growth factor secreted from liver sinusoidal endothelial cells
Source: Sci Rep. 2020 Sep 1;10:14349. doi: 10.1038/s41598-020-71453-5 (PMC7462976; doi:10.1038/s41598-020-71453-5)
Supplement: Supplementary file 1 — Supplementary Information 1. [file 41598_2020_71453_MOESM1_ESM.pdf]

Modulation of Hepatitis B virus infection by epidermal growth factor secreted from liver sinusoidal endothelial cells

Shin-Wei Chen[1], Misao Himeno[1], Yuta Koui[1], Masaya Sugiyama[2], Hironori Nishitsuji[2], Masashi Mizokami[2], Kunitada Shimotohno[2], Atsushi Miyajima[1] and Taketomo Kido[1*]

[1]Institute for Quantitative Biosciences, The University of Tokyo

1-1-1 Yayoi, Bunkyo-ku, Tokyo 113-0032, Japan

[2]Genome Medical Science Project, National Center for Global Health and Medicine

1-7-1 Kohnodai, Ichikawa, 272-8516, Japan

Supplementary Table 1. List of quantitative PCR primers for mouse genes

gene	Left Primer	Right Primer
Stab2	TGTCCAGACGGCTACATCAA	CCAGGGATATCCAGGACGTA
Lyve1	CCTCCAGCCAAAAGTTCAAA	TCCAACACGGGGTAAAATGT
Pecam1	CTGGTGCTCTATGCAAGCCT	AGTTGCTGCCCATTCATCAC
F8	TCATGTATAGCCTGGATGGGA	GATGAGTCCACATTGCCAAA
Ngfr	GTGTGCGAGGACACTGAGC	GGGGGTAGACCTTGTGATCC
Desmin	GTGAAGATGGCCTTGGATGT	CTCGGAAGTTGAGAGCAGAGA
Hgf	CCTGACACCACTTGGGAGTA	CTTCTCCTTGGCCTTGAATG
Actb	TTCTTTGCAGCTCCTTCGTT	ATGGAGGGGAATACAGCCC

Supplementary Table 2. List of quantitative PCR primers and probes for HBV detection

	Primer/Probe
HBSF2	CTTCATCCTGCTGCTATGCCT
HBSR2	AAAGCCCAGGATGATGGGAT
cccDNA F7	TCCCCGTCTGTGCCTTCTC
cccDNA R7	GCACAGCTTGGAGGCTTGA
cccDNA P7	FAM- CCGTGTGCACTTCG

Supplementary Table 3. List of primary and secondary antibodies used for immunocytochemistry analysis and FCM analysis of mouse and human cells

Primary Antibody	Supplier
LAMP2	abcam (ab25631)
EGFR	cell signaling (D38B1)
Myc	abcam (ab206486)
Stabilin2	Nonaka et al.
LNGFR	Miltenyi biotec (REA648)

Secondary Antibody	Supplier
Alexa Fluor 488 anti-Rabbit IgG	Invitrogen (A32790)
Alexa Fluor 555 anti-Mouse IgG	Invitrogen (A21424)

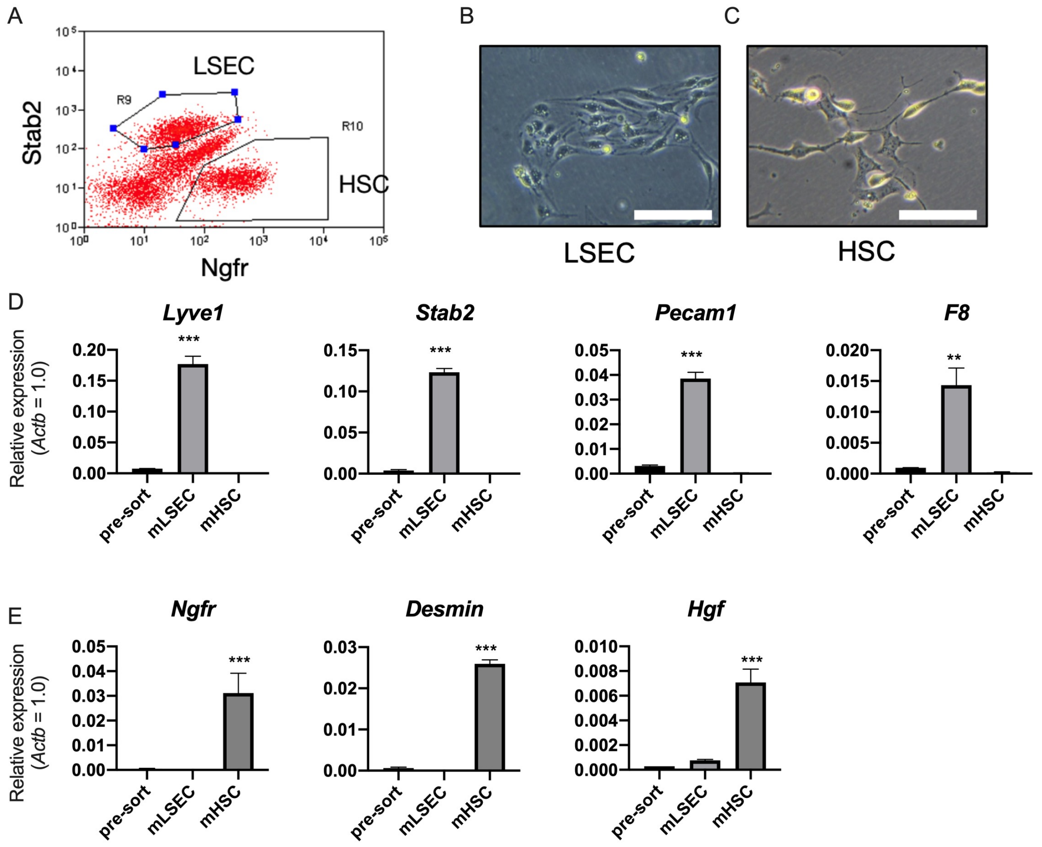

Supplementary Fig. 1. Identification and characterization of fetal mouse LSECs and HSCs.

(A) Flow cytometric analysis of fetal mouse liver cells at E14.5.

(B) Primary culture of Stab2^{+} cells. Scale bar, 100 μm.

(C) Primary culture of Ngfr^{+} cells. Scale bar, 100 μm.

(D) Expression levels of LSEC markers in pre-sorting cells (pre-sort), Stab2^{+} cells (LSECs), and Ngfr^{+} cells (HSCs). The results are shown as the mean ± SEM of 3 independent experiments. n = 3 in each group. The significant difference was determined

in each group compared with the control group. ***p < 0.001.

(E) Expression levels of HSC markers in pre-sorting cells (pre-sort), Stab2$^+$ cells (LSECs) and Ngfr$^+$ (HSCs) cells. The results are shown as the mean ± SEM of 3 independent experiments. The significant difference was determined in each group compared with the control group. ***p < 0.001.

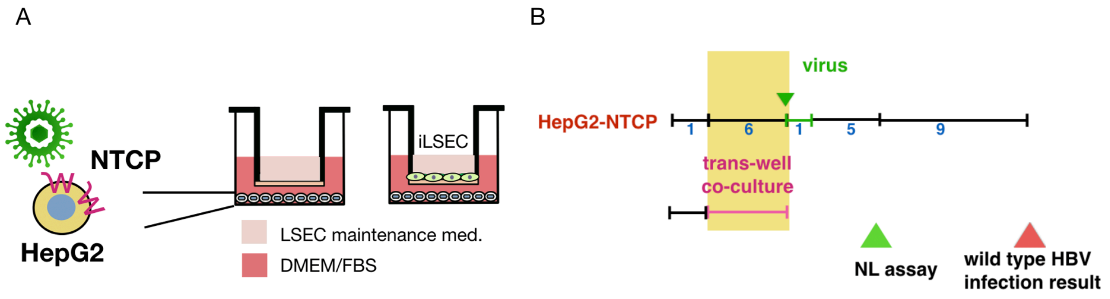

Supplementary Fig. 2.

HBV infection to HepG2-NTCP cells co-cultured with iPSC-derived LSECs in the trans-well system.

(A) Experimental design of the trans-well co-culture system of HepG2-NTCP cells with iPSC-derived LSECs.

(B) Experimental design of HBV infection using HBV/NL and wild type HBV in the trans-well co-culture system

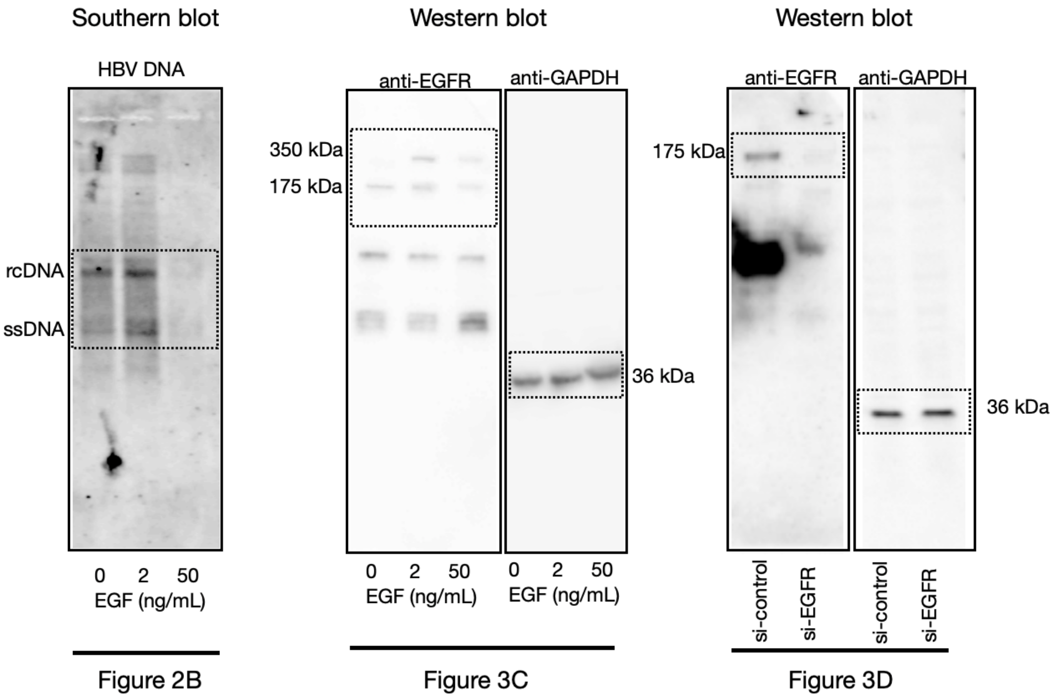

Supplementary Fig. 3.

The images of full-length blots in figures

(Left) Southern blot analysis in Figure 2B, (Middle) Western blot analysis in Figure 3C, (Right) Western blot analysis in Figure 3D.
